# Serotoninergic Modulation of Phototactic Variability Underpins a Bet-Hedging Strategy in *Drosophila melanogaster*

**DOI:** 10.3389/fnbeh.2021.659331

**Published:** 2021-04-16

**Authors:** Indrikis A. Krams, Tatjana Krama, Ronalds Krams, Giedrius Trakimas, Sergejs Popovs, Priit Jõers, Maris Munkevics, Didzis Elferts, Markus J. Rantala, Jānis Makņa, Benjamin L. de Bivort

**Affiliations:** ^1^Institute of Ecology and Earth Sciences, University of Tartu, Tartu, Estonia; ^2^Department of Zoology and Animal Ecology, Faculty of Biology, University of Latvia, Riga, Latvia; ^3^Department of Psychology, University of Tennessee, Knoxville, TN, United States; ^4^Department of Biotechnology, Daugavpils University, Daugavpils, Latvia; ^5^Chair of Plant Health, Estonian University of Life Sciences, Tartu, Estonia; ^6^Institute of Biosciences, Vilnius University, Vilnius, Lithuania; ^7^Institute of Molecular and Cell Biology, University of Tartu, Tartu, Estonia; ^8^Department of Botany and Ecology, Faculty of Biology, University of Latvia, Riga, Latvia; ^9^Department of Biology, Section of Ecology, University of Turku, Turku, Finland; ^10^Department of Artificial Intelligence and Systems Engineering, Riga Technical University, Riga, Latvia; ^11^Department of Organismic and Evolutionary Biology, Harvard University, Cambridge, MA, United States

**Keywords:** adaptive strategies, *Drosophila melanogaster*, phototaxis, serotonin, variation

## Abstract

When organisms’ environmental conditions vary unpredictably in time, it can be advantageous for individuals to hedge their phenotypic bets. It has been shown that a bet-hedging strategy possibly underlies the high inter-individual diversity of phototactic choice in *Drosophila melanogaster*. This study shows that fruit flies from a population living in a boreal and relatively unpredictable climate have more variable variable phototactic biases than fruit flies from a more stable tropical climate, consistent with bet-hedging theory. We experimentally show that phototactic variability of *D. melanogaster* is regulated by the neurotransmitter serotonin (5-HT), which acts as a suppressor of the variability of phototactic choices. When fed 5-HT precursor, boreal flies exhibited lower variability, and they were insensitive to 5-HT inhibitor. The opposite pattern was seen in the tropical flies. Thus, the reduction of 5-HT in fruit flies’ brains may be the mechanistic basis of an adaptive bet-hedging strategy in a less predictable boreal climate.

## Introduction

Life in the natural environment exposes organisms to a number of abiotic and biotic factors. The stability of environmental conditions differs across latitudes, geographic locations and time, resulting in diverse patterns of fluctuation in ecological interactions, population sizes, sex and age ratios, parasite burdens, range shifts and extinction. To predict the direction and magnitude of responses to fluctuating environments, it is important to understand the strategies that facilitate the survival of organisms under environmental change (Merilä and Hendry, 2014; Chirgwin et al., 2015; Trakimas et al., 2019). One such strategy is phenotypic plasticity, in which organisms adjust their phenotypes in accordance with their current development conditions. Although phenotypic plasticity is a versatile strategy and can potentially apply to behavioral, morphological, physiological or biochemical traits, there are constraints to plasticity such as limits on the speed with which a phenotype can change in response to a fluctuating environment (Folguera et al., 2010; Murren et al., 2015). To survive and reproduce in fluctuating environments, individuals might use also another strategy: evolving by natural selection with the changing environment (Langerhans and DeWitt, 2004). This is one of the most powerful means to track ongoing biotic and abiotic fluctuations such as climate change.

Some organisms live in conditions where ambient temperature, precipitation, food resources, and other important selection factors occur in highly variable patterns (Siepielski et al., 2017). In these climates, the time windows favorable for reproductive cycles and offspring development can be short. Therefore, there is a pressure on individuals to start the reproductive season as soon as suitable conditions arrive. However, if all the individuals invest in reproduction at the first possible moment, the entire population is vulnerable to extinction if the favorable conditions unexpectedly cease. Therefore, it can be useful if individuals in a population, hedge their bets and some of them delay the onset of reproduction, otherwise no offspring result from the population experiencing these false starts. There are three bet-hedging strategies: Conservative bet-hedging (always play it safe), diversified bet-hedging (do not put all eggs in one basket) and adaptive coin flipping (random selection of the available strategies; Olofsson et al., 2009). Bet-hedging is potentially adaptive for many additional traits, including behavior, when the selective pressures of the environment fluctuates strongly (Kain et al., 2015).

The fruit fly *Drosophila melanogaster* is one of the most studied model organisms in biology. Flies appear to use bet-hedging as an adaptive response to environmental uncertainty, exhibiting variation in their bodily development (Krams et al., 2020) and light- and temperature-preference behaviors (Kain et al., 2015). Phototactic diversity appears to be a form of fly personality and mechanistic experiments identified the neurotransmitter serotonin (5-HT) as a suppressor of phototactic variability (Kain et al., 2012). Evolutionary modeling suggested that a bet-hedging strategy underlies the observed inter-individual diversity of phototactic and thermotactic choice. Specifically, it was suggested that flies from areas with large seasonal weather fluctuations should have greater behavioral unpredictability than fruit flies from warmer and more predictable climates (Kain et al., 2015). However, these predictions have never been tested using flies from different climate zones. In this study, we tested whether flies from northern Europe, adapted to a temperate climate with strong fluctuations, exhibit less predictable phototactic biases than those from southern Europe and tropical Africa. To assess whether potential variation in bet-hedging between these populations depends on serotonin, we tested the phototactic behavior of fruit flies collected in Finland and Kenya with and without treating them with tryptophan (5-HTP, a precursor of 5-HT synthesis) and α-methyl-tryptophan (αMW, a serotonin-synthesis inhibitor), pharmacological agents with well-established effects on fruit fly behavior and serotonin levels (Dasari et al., 2007; Dierick and Greenspan, 2007; Neckameyer, 2010; Majeed et al., 2016; Ries et al., 2017; Hu et al., 2020).

The northernmost record of overwintering populations of *D. melanogaster* is from southwest Finland (Keller, 2007), while this cosmopolitan species (Markow, 2015) likely originated in the tropical region including Kenya (David and Capy, 1988; Lachaise et al., 1988; Baudry et al., 2004; Keller, 2007). We predicted that flies from the northern climate would be less predictable in their phototactic biases because they have adapted to a rapidly fluctuating environment with a bet-hedging strategy. This could be achieved by a reduction of 5-HT in their brains, in which case feeding these flies 5-HTP should decrease the variability of their phototactic biases. Conversely, the brains of Kenyan flies might contain more serotonin, in which case adding αMW should increase the unpredictability of their phototactic biases. Variability of phototactic biases was assessed by measuring the variability beyond expectation (VBE), which compares the observed variation to the variation expected due to sampling error (Kain et al., 2015). As the VBE is a nonparametric parameter and sensitive to outliers, it has a low signal-to-noise ratio. We have overcome this disadvantage using large sample sizes in each experimental group and nonparametric analyses.

## Materials and Methods

### Insects

We studied the phototactic behavior of fruit flies sampled from wild populations near Mombasa (Kenya) and between Turku and Tampere (Finland). Local wild flies were caught using banana and yeast baited traps or collecting the pupae from compost piles in April 2017, in Kenya and in August 2017 in Finland. In either country, we collected the flies from five locations separated by a 5-km distance at least.

For behavior experiments, parental flies were kept in vials for 24-h to oviposit. When their progeny enclosed, they were removed in the first 5–7 h post-eclosion, anesthetized, and separated by sex. In this study, we tested flies grown on Formula 4–24 instant *Drosophila* media (item #173202, Carolina Biological Supply Company, Burlington, NC, USA).

### Phototaxis Equipment

We studied the variability of phototaxis behavior in F1 flies. The FlyVac apparatus allowed us to measure the startled phototaxis behavior of many individual fruit flies simultaneously (Kain et al., 2012). The operational details of FlyVac are detailed elsewhere (Kain et al., 2012). In brief, FlyVac is an instrument for the rapid quantification of phototaxis behavior. Up to 32 individual fruit flies were loaded into separate phototaxis modules, each consisting of a phototactic T-maze in which the fly could choose between a light [an illuminated light-emitting diode (LED)] and dark stimulus (a non-illuminated LED). Both branches of the T-maze are equipped with an LED but only one LED is illuminated, at random, in each trial.

To begin a phototaxis session, individual flies are aspirated from its culture vial into the vertical start tube of the T-maze. After insertion, a fly climbs upward through the vertical tube of the T-maze under negative geotaxis until it reaches the choice point of the T-maze. Upon making a choice by entering one of the corridors of the T-maze, the fly is detected by an optical interrupter. This triggers recording the direction of the choice done with respect to the direction of the illuminated stimulus LED and opens a vacuum to pull the fly back into the start tube. In each trial, one LED out of two is lit at random. After completing 40 trials, the phototaxis module is deactivated and the flies are simply contained until removal. In the event that a fly does not complete 40 trials within several hours, that fly is removed from the module and further analyses. Before the trials, we have checkered whether the FlyVac apparatus itself was not affecting behavior. We have performed a long series of assays with two LEDs on and with two LEDs off. In both cases, the resulting distributions are statistically indistinguishable from the random binomial distribution.

### Groups and Drug Treatments

We had three experimental groups per geographic location: flies (males and females) grown without any drugs, flies grown on food supplemented with 5-HTP and flies grown on food supplemented with αMW (Dasari et al., 2007; Dierick and Greenspan, 2007; Neckameyer, 2010; Majeed et al., 2016; Ries et al., 2017; Hu et al., 2020). Drugs were dissolved in Formula 4–24 instant *Drosophila* media. For the drug-feeding, F0 flies laid eggs in drug-containing media. Upon eclosion, adult F1 flies were assayed on days 2–3. The drug stock solutions were vortex-mixed and added to food powder. The final concentration of 5-HTP was 50 mM and the final concentration of αMW was 20 mM (Huber and Ronchetti, 2009; Kain et al., 2012).

### Statistics

We first fitted the Poisson (with log link) generalized linear model (GLM) using positive light-choice count as a dependent variable and geographic location (Finland, Kenya), treatment groups (control, 5-HTP, αMW) and sex as predictors with interaction (sex × treatment groups). After determining that sex was not a significant factor, we excluded it and fitted the final GLM using geographic location, group and an interaction between geographic location and treatment group. We assessed the variation of phototactic choices by calculating the variability beyond expectation (VBE; Kain et al., 2015). This metric is equal to log2(μADobs/μADexp), where μADobs indicates the mean absolute deviation of the data from their median and μADexp is the equivalent value calculated from the binomial distribution corresponding to the sampling error expected if all individuals made light choices with identical probabilities (Huber and Ronchetti, 2009). Standard errors for VBE were calculated by bootstrap resampling (5,000 replicates) of individual flies. Pairwise comparisons of light-choice probability (LCP) between groups were made using Mann–Whitney *U* tests.

## Results

### Light-Choice Probability (LCP)

**Figure 1:**
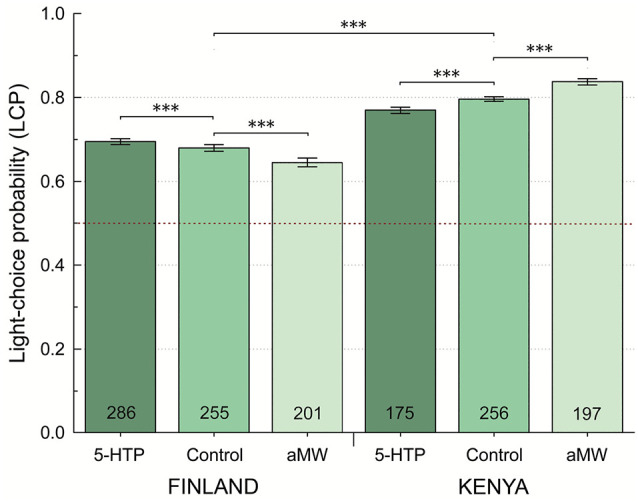
Light-choice probability (LCP) by site and pharmacological treatment. 5-HTP, Tryptophan, a precursor of serotonin synthesis; αMW, α-methyl-tryptophan, a serotonin-synthesis inhibitor. Error bars are ±1 SE, calculated by bootstrap resampling; ***P < 0.001.

We found that female and male flies did not differ significantly in their LCP (fraction of choices toward the illuminated LED) either in Finland or Kenya (Poisson GLM with log link, Wald Chisq_1_ = 0.66, *P* > 0.4) and, therefore, we pooled sexes in the further analyses of LCP. We found that geographic location was a significant predictor of light-choice probability (Poisson GLM with log link, Wald Chisq_1_ = 290.8, *P* < 0.0001). Flies were photopositive both in Kenya and Finland, choosing the light 80% and 68% of the time (Table 1; Figure 1), respectively. Kenyan flies were found to be significantly more photopositive than Finnish flies (*P* < 0.001; Table 1; Figure 1). While the main effect of treatment group on light-choice was not significant (Poisson GLM with log link, Wald Chisq_2_ = 0.449, *P* = 0.8), however, there was significant interaction between geographic location and treatment group (Poisson GLM with log link, Wald Chisq_2_ = 40.93, *P* < 0.0001). Feeding Kenyan flies, αMW (a serotonin synthesis inhibitor) increased their LCP significantly while feeding 5-hydroxytryptophan, a serotonin precursor (5-HTP) reduced LCP (Table 1; Figure 1). In contrast, feeding Finnish flies αMW reduced LCP, while 5-HTP increased their LCP. However, LCP of Finnish flies fed 5-HTP was still lower than the LCP of Kenyan flies (Table 1; Figure 1).

**Table 1:** Descriptive statistics of light-choice probability (LCP) and variability beyond expectation (VBE); ±SE were based on bootstrap resampling (5,000 replicates).

			Light choice	Variability beyond
			probability	expectation
Geographic location	Treatment	*n*	(LCP) ± SE	(VBE) ± SE
Finland	5-HTP	286	0.695 ± 0.00665	0.5278 ± 0.06943
Finland	Control	255	0.6798 ± 0.00779	0.7225 ± 0.0703
Finland	αMW	201	0.6454 ± 0.00977	0.8394 ± 0.07307
Kenya	5-HTP	175	0.7673 ± 0.00745	0.5458 ± 0.085
Kenya	Control	256	0.7964 ± 0.00564	0.3839 ± 0.07685
Kenya	αMW	197	0.8379 ± 0.00663	0.6392 ± 0.07468

### Variability Beyond Expectation (VBE)

**Figure 2:**
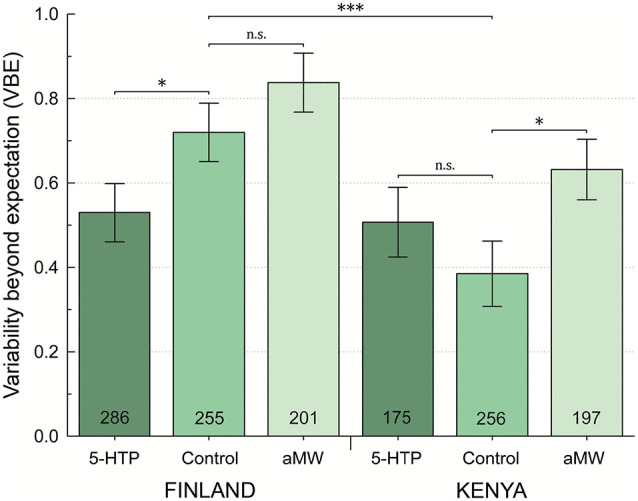
Behavioral variability beyond statistical expectation (VBE) by site and pharmacological treatment. On a log2 scale, VBE = 0 indicates no excess variability, and VBE = 1 indicates twice as much variability as would be expected by chance alone. Error bars are ±1 SE, calculated by bootstrap resampling; ^n.s.^*P* > 0.05, *P < 0.05, ***P < 0.001 represent one-tailed significance.

Female and male flies had similar among-individual phototactic variability, as measured by VBE, in both Finland and Kenya (all bootstrapped distributions, *P* > 0.05) and sexes were pooled in the further analyses of VBE. Finnish fruit flies had significantly higher VBE than flies in Kenya (*P* < 0.001; Table 1; Figure 2). Feeding αMW did not affect the VBE of Finnish flies, whereas adding 5-HTP to their food significantly suppressed VBE (*P* = 0.023). In Kenyan flies, feeding αMW significantly increased VBE, while 5-HTP did not affect their VBE (Table 1; Figure 2). Importantly, feeding 5-HTP made VBE of Finnish flies similar to VBE of Kenyan flies (Table 1; Figure 2).

## Discussion

In this study, we examined light-choice probability and the variation beyond expectation of the light choice probability in fruit flies from tropical and boreal climates. Although all flies were raised and tested in identical conditions, Kenyan fruit flies originated from areas with stable and predictable climate, while Finnish fruit flies originated in the northernmost limits of the species’ distribution range, a zone characterized by an intensely fluctuating climate. We found that flies from the southern climate and were more strongly attracted to light (higher LCP), and more consistent across individuals (lower VBE) than flies from the northern climate. Conversely, flies from the higher latitude site were less attracted to light and less consistent across individuals. Bet-hedging theory predicts that higher phenotypic diversity may be adaptive under conditions of less predictable climate. Kain et al. (2015) developed a computational model suggesting that this hypothesis is plausible specifically with respect to *Drosophila* light-choice behavior. The results of this study provide empirical evidence in support of this model using fly strains caught at geographic sites with differential climatic variability (Akhund-Zade et al., 2020).

Previous work shows that genetic variation within lab strains likely cannot account for the variation of phototactic responses (Kain et al., 2012). In contrast, VBE varied significantly between flies collected in eastern Africa and northern Europe, suggesting that genetic factors underlie differences in the magnitudes of variation. A genetic basis for variability has been found in several other *Drosophila* behaviors including locomotor bias (Ayroles et al., 2015) and odor preference (Honegger et al., 2019). With respect to phototaxis, it was found that the gene *white* has an important role as an importer of metabolic precursors of serotonin (Kain et al., 2012). In this study, we significantly decreased VBE of Kenyan flies by feeding them αMW, an inhibitor of serotonin biosynthesis. Feeding Finnish fruit flies 5-HTP, a serotonin biosynthesis precursor, significantly reduced their VBE. Thus, manipulations to reduce serotonin levels in Kenyan flies and increase them in Finnish flies made VBE of these groups statistically similar. Notably, feeding Kenyan flies 5-HTP did not significantly reduce the VBE of these flies. Likewise, feeding Finnish flies αMW did not increase their VBE. These results suggest that a possible ceiling effect in Kenyan flies renders them insensitive to additional serotonin. Conversely a floor effect in Finnish flies may explain their insensitivity to serotonin inhibitors (Lam, 1996). However, we cannot exclude the possibility that the 5-HT-related effects on light choice and the variability of choices are also due to independent mechanisms. For example, independent genetic effects on the mean and variability of light choice were observed when the *white* gene was muted (Kain et al., 2012).

We found that Finnish flies exhibited high VBE for phototaxis, similarly to what was observed in a laboratory strain, *D. melanogaster* *w*^1118^ (Kain et al., 2012). These flies have white eyes (Morgan, 1910; Green, 1996) due to a mutation in the gene *white*, which is a central part of the eye-pigmentation pathway (Ferreiro et al., 2018). This gene encodes White, an ATP binding cassette transporter (Pepling and Mount, 1990), that heterodimerizes with either Brown or Scarlet proteins, encoded by brown and scarlet genes to transport guanine or 5-HTP, respectively. In neurons, these transporters contribute to the biosynthesis of amines. It has been shown that *white* mutants have significantly reduced levels of the neurotransmitters serotonin (up to five times lower), dopamine, and histamine (Borycz et al., 2008; Sitaraman et al., 2008), especially in glia and neurons of the brain (Borycz et al., 2008). These diminished concentrations of the neurotransmitters in *white* mutants (Borycz et al., 2008; Sitaraman et al., 2008) have multiple consequences on a variety of neurological phenotypes affecting male courtship behavior (Zhang and Odenwald, 1995; Anaka et al., 2008; Lee et al., 2008), anesthesia resistance (Campbell and Nash, 2001), aggressive behavior (Hoyer et al., 2008), spatial learning and olfactory learning (Diegelmann et al., 2006; Anaka et al., 2008; Sitaraman et al., 2008), duration of periods of locomotion recovery following anoxia (Xiao and Robertson, 2016), sensitivity to ethanol (Chan et al., 2014), sensitivity to certain tactile stimuli (Titlow et al., 2014) and propensity to retinal degeneration (Ferreiro et al., 2018). Although Finnish fruit flies have normal red eyes, they displayed average light preference (68%) and VBE (0.72) similar to the values seen in *white* mutants. For example, *w^1118^* mutants chose light 61% of the time and their VBE is ~0.87, values which are closer to those in Finnish flies than, for example, flies of the standard lab wild type strain Canton-S (76% and 0.56, respectively; Kain et al., 2012).

Common factors may be responsible for the behavioral metrics of Finnish and *w^1118^* flies. We found that 5-HTP significantly affected VBE of Finnish flies, while it had no effect on VBE of Kenyan flies. The same pattern was observed in *w^1118^* and Canton-S flies, respectively (Kain et al., 2012). This suggests that the brains of African flies contain a higher concentration of serotonin, perhaps because their food sources are more diverse and may contain more metabolite precursors than Finnish flies. Tryptophan, a serotonin precursor, is an essential amino acid because animals cannot synthesize it but instead must obtain it through their diet. While African flies often enjoy the availability of different fruits, mushrooms, sap fluxes year-round, Finnish fruit flies have much shorter summer season in general, with reduced availability of rotting and decaying fruits and mushrooms in particular (Sardeshpande and Shackleton, 2019). The depletion of tryptophan from the diet has been used to assess brain serotonergic function in humans (Lam, 1996). This procedure is capable of rapidly lowering brain tryptophan levels in human patients by over 80% within just a few hours (Young et al., 1985), which may have immediate effects on depression patients (Smith et al., 1997; Neumeister et al., 1998) such as deviations from normal behavior and lowered food intake (Rantala et al., 2018, 2019).

Theory predicts that the relative stability of the local climate in Kenya should favor heritable and lower variability phototactic preferences, i.e., a strategy with less stochastic bet-hedging (Hopper, 1999). In such strategies, the current mean phenotype always lags environmental fluctuations, because evolution by natural selection is not instantaneous. In predictable environments, the penalty for this lag is minimized. By contrast, Finnish flies showed significantly more variable phototactic preferences, suggestive of an adaptive bet-hedging and consistent with previous modeling of bet-hedging in thermal preference behavior (Kain et al., 2015). Interestingly, adaptations for heat resistance have the potential to improve cold resistance (Condon et al., 2014). This shows that adaptations to extreme temperatures improve not only the ability to withstand a particular deviation from mean temperatures, but also the magnitude of temperature variation. Moreover, the ability to tolerate extreme temperatures is improved in populations that evolve in fluctuating environments relative to when populations are exposed to a stable increase of high temperatures (Condon et al., 2014; Tobler et al., 2015). The high VBE of Finnish flies, which in the wild may result in a variety of thermal experiences, may serve as parallel adaptation to life in relatively unpredictable thermal and visual environments, leading flies to find conspecifics, breed and oviposit in a variety of conditions, rather than wait for specific optimal conditions that might not arrive in a particular season.

In a population utilizing a bet-hedging strategy, individuals exhibiting a wide variety of preferences are born continuously across a season. If the summer is cooler, spring-adapted individuals will survive, while summer-adapted flies will survive if the summer is hot and long (Bergland et al., 2014). Kawecki (2000) has suggested that the phenotypic expression of genetic variation can be suppressed, and heritability reduced under fluctuating selection. Dynamic modulation of variability-suppressing serotonin is a potential mechanism to tune the canalization of the phototactic phenotype. To test this possibility, one could measure VBE and 5-HT concentration in the brains of flies, born during hot and cool summers near the northernmost areas of their distribution ranges. Our results suggest that plastic responses to environmental differences, which is another major strategy for dealing with environmental heterogeneity, is not a likely explanation for the observed differences between African and European flies. The flies of both populations were grown under identical conditions and we are not aware of any environmental fluctuations to which a plasticity strategy could respond.

While Kain et al. (2012) observed significant effects of 5-HTP on the VBE of flies, this treatment did not show any influence on light-choice probability in their study. However, we found significant effects of feeding 5-HTP and αMW on LCP, which depended on the origin of the strain. In addition, we observed effects on LCP of line origin, with Kenyan flies ~10% more photopositive. In Finnish flies, feeding 5-HTP did not affect LCP, while feeding αMW significantly reduced it, which was the opposite of what we observed in the case of VBE in these flies. Feeding 5-HTP significantly lowered LCP and feeding αMW significantly raised LCP in Kenyan flies. Thus, 5-HTP decreased the light choice probability in Kenyan flies and did not affect it in Finnish flies. Importantly, Kenyan flies on control media chose the light more often than Finnish flies on control media. Kenyan flies are likely to have a higher concentration of 5-HT in the brains, at least when fed natural diets.

It is possible that differential levels of serotonin do not explain the mean LCP of these strains, since serotonin or its precursor 5-HTP have been previously reported to decrease photopositivity in larval bryozoans (Pires and Woollacott, 1997). There may also be genetic background by serotonin-exposure effects. However, dopamine was previously reported to increase light choice (Pires and Woollacott, 1997). *White* mutants have reduced concentrations of dopamine in the brain (Borycz et al., 2008; Sitaraman et al., 2008) and if the neuromodulatory state of Finnish flies mirrors that of *white* mutants, they may also have lower dopamine levels. This in turn might explain their lower LCP, while lower serotonin could explain their higher VBE. Kain et al. (2012) did not find any effect of dopamine drugs on VBE or LCP of different strains of fruit flies including *white* mutants. However, it has been shown that dopamine affects the production and release of melatonin (González et al., 2012), a key driver of biological rhythm (Arendt and Skene, 2005). Melatonin production might be disrupted in the brains of Finnish flies to ensure activity during long summer days at high latitudes indicating that dopamine of boreal fruit flies, especially the receptor subtypes and the density of receptors deserve a special attention in future research.

Importantly, 5-HT is a precursor of melatonin (Richter et al., 2002), and 5-HT is also regarded as a substance affecting physiological rhythms according to the light–dark cycle in invertebrates (Hardeland and Poeggeler, 2003). Kenyan and Finnish flies likely have different diurnal rhythms and sleep patterns: While there is a relatively regular day/night cycle in the tropical zone, Finnish flies enjoy never-ending daylight for up to two months at high latitudes. This may affect their serotonergic neural regulation because melatonin may be in low demand and not metabolized much during the northern summer, perhaps allowing the accumulation of elevated 5-HT in neural tissues in summer. Besides a leading role of melatonin in the determination of sleep/wake cycles, it is also a potent antioxidant with a proposed role in immune function in invertebrates (Tan et al., 2009). The suppression of nocturnal production of melatonin has detrimental effects on antioxidant systems of organisms (Jones et al., 2015) which may facilitate the bet-hedging strategy of invasive species at high latitudes.

Flies that follow a bet-hedging strategy might only attain environmental conditions well-matched to their behavioral biases if they live through long periods of poorly matched conditions. Thus, there is likely an interplay between generation/lifespan length and the timescale of environmental fluctuations. Indeed, modeling suggests that bet-hedging is an adaptive response to environmental fluctuations at specific timescales roughly corresponding to the lifespan (Krams et al., 2020). Melatonin is an antioxidant, and may lengthen the lifespan of flies (Terán et al., 2012). Thus, it has the potential to affect evolutionary behavioral strategies both directly through the neuromodulatory state, but also indirectly through an effect on lifespan. These hypotheses call for precise measurements of 5-HT, melatonin, dopamine and behavior in fruit flies across the season and the south-north gradient of their distribution range.

## Conclusions

Proving that phenotypic variability reflects a bet-hedging strategy is a tall order. A formal approach to this question (Simons, 2011), stipulates six increasingly convincing categories of evidence for bet-hedging: (1) description of a potential bet-hedging trait, (2) measured variability of the environmental characteristics pertinent to the bet-hedging strategy, (3) genotype-specificity of variability or a genetic mechanism underlying it, (4) identified fitness consequences of phenotypic variations, (5) experiments showing that bet-hedging provides a geometric-mean fitness advantage in a fluctuating environment, and (6) an empirical match to the predicted optimal magnitude of phenotypic fluctuation in response to environmental fluctuation. With respect to phototaxis in flies, this work provides evidence in the first three categories. Flies vary in their phototactic preferences, and this may reflect a bet-hedging strategy in response to fluctuations in luminance and thermal fluctuations, which are greater in Finland than in Kenya. Fly strains from these sites exhibit lab-stable differences in variability, consistent with a genotypic basis to bet-hedging (Category 3). We speculate that individual phototactic preferences may have fitness consequences (Category 4) through temperature-dependent effects on life history (Kain et al., 2015; Akhund-Zade et al., 2020) or differential access to environmental resources. However, establishing this link empirically and acquiring evidence for bet-hedging in Categories 5 and 6 will require additional studies.

## Data Availability Statement

The raw data supporting the conclusions of this article will be made available by the authors, without undue reservation.

## Author Contributions

IK, TK, RK and BB conceived and designed the study. TK, RK, IK, GT, SP, PJ, MM and MR performed the study, collected and extracted data. GT, DE, RK and IK analyzed the data. JM, SP and IK built the equipment. BB, TK, MR, PJ, SP, JM and MM participated in data analyses, results interpretation, and drafting the manuscript. All authors contributed to the article and approved the submitted version.

## Conflict of Interest

The authors declare that the research was conducted in the absence of any commercial or financial relationships that could be construed as a potential conflict of interest.
